# Stating Appointment Costs in SMS Reminders Reduces Missed Hospital Appointments: Findings from Two Randomised Controlled Trials

**DOI:** 10.1371/journal.pone.0137306

**Published:** 2015-09-14

**Authors:** Michael Hallsworth, Dan Berry, Michael Sanders, Anna Sallis, Dominic King, Ivo Vlaev, Ara Darzi

**Affiliations:** 1 Centre for Health Policy, Imperial College London, London, United Kingdom; 2 The Behavioural Insights Team, 33 Greycoat Street, London, United Kingdom; 3 Department of Health, Richmond House, 79 Whitehall, London, United Kingdom; 4 Harvard Kennedy School, 79 John F. Kennedy Street Cambridge, MA, United States of America; 5 Public Health England, Skipton House, 80 London Road, London, United Kingdom; University of California San Diego, UNITED STATES

## Abstract

**Background:**

Missed hospital appointments are a major cause of inefficiency worldwide. Healthcare providers are increasingly using Short Message Service reminders to reduce ‘Did Not Attend’ (DNA) rates. Systematic reviews show that sending such reminders is effective, but there is no evidence on whether their impact is affected by their content. Accordingly, we undertook two randomised controlled trials that tested the impact of rephrasing appointment reminders on DNA rates in the United Kingdom.

**Trial Methods:**

Participants were outpatients with a valid mobile telephone number and an outpatient appointment between November 2013 and January 2014 (Trial One, 10,111 participants) or March and May 2014 (Trial Two, 9,848 participants). Appointments were randomly allocated to one of four reminder messages, which were issued five days in advance. Message assignment was then compared against appointment outcomes (appointment attendance, DNA, cancellation by patient).

**Results:**

In Trial One, a message including the cost of a missed appointment to the health system produced a DNA rate of 8.4%, compared to 11.1% for the existing message (OR 0.74, 95% CI 0.61–0.89, P<0.01). Trial Two replicated this effect (DNA rate 8.2%), but also found that expressing the same concept in general terms was significantly less effective (DNA rate 9.9%, OR 1.22, 95% CI 1.00–1.48, P<0.05). Moving from the existing reminder to the more effective costs message would result in 5,800 fewer missed appointments per year in the National Health Service Trust in question, at no additional cost. The study’s main limitations are that it took place in a single location in England, and that it required accurate phone records, which were only obtained for 20% of eligible patients. We conclude that missed appointments can be reduced, for no additional cost, by introducing persuasive messages to appointment reminders. Future studies could examine the impact of varying reminder messages in other health systems.

**Trial Registration:**

Controlled-Trials.com 49432571

## Introduction

“Did Not Attends” (DNAs) occur when a patient unexpectedly fails to attend an appointment [1]. In 2012–13, around 5.5 million National Health Service (NHS) outpatient appointments were missed in England (9.3% of the total) [2]. Whilst it is difficult to establish the exact financial impact of DNAs, a recent estimate claimed that missed first outpatient appointments cost the NHS up to £225 million in 2012–13 [3]. DNAs lead to worse care for patients, the inefficient use of staff, and increased waiting times [4] [5]. Hospitals may also engage in complex compensating behaviours, such as overbooking, which introduce problems of their own [6].

Survey evidence indicates that the main reason for a DNA is the patient forgetting their appointment (although we recognise that other factors, such as dissatisfaction with standards of care, are also important) [7] [8] [9]. Attention has therefore focused on the effective use of reminders. Systematic reviews have found that telephone and Short Message Service (SMS) reminders significantly reduce non-attendance, although a recent Cochrane Review judged that the quality of existing evidence is only of low to moderate quality [10] [11]. There is also evidence that SMS reminders are more cost-effective than telephone reminders [12]. The use of SMS reminders is widespread in the NHS: for example, approximately five million were sent via NHS Mail per monthin 2013 and 2014 [13] [14].

There is, however, no evidence on whether or how the effectiveness of these reminders is affected by their content [10]. Other studies have shown that compliance can be increased by even small changes to the phrasing of messages [15] [16]. Therefore it is possible that changes to reminder wording could lead to savings and improved patient care for no additional cost. Given the scale of SMS usage, this is a significant opportunity for service improvement. Accordingly, we undertook two randomised controlled trials to test the impact of rephrasing appointment reminders on DNA rates amongst hospital outpatients in a London NHS Trust.

## Ethics and Registration

Ethical approval was obtained from a Research Ethics Committee of the NHS National Research Ethics Service (NHS REC 13/NW/0508). Individual informed consent for participation was not obtained, as approved by the Committee. Obtaining consent would introduce burdens to the patient (larger than the intervention itself); obtaining informed consent would cause serious practical problems that would undermine the trial results (particularly for the control group); and the risk of harm was low, since the intervention merely consisted of small modifications to existing routine processes.

This trial was registered on ISRCTN after it had commenced (ISRCTN 49432571 http://www.controlled-trials.com/ISRCTN49432571/). This is because the study team received guidance in its REC approval letter that there was a period of six weeks from commencement within which to register the trial. All other SMS trials the authors are involved in are registered.

## Trial One Methods

### Trial design

The trial took place in an NHS Trust (Barts Health) that used an SMS provider (iPlato) to send outpatient appointment reminders. Participants were all patients with a valid mobile telephone number and an outpatient appointment in November and December 2013 and January 2014 in at least one of five specialities: rheumatology, ophthalmology, gastroenterology, neurology, and cardiology. These specialities were chosen because they were not currently the subject of any other initiatives to reduce DNAs, apart from the SMS reminders already in use.

Appointments were randomly allocated to one of four reminder messages, which were issued five days in advance. Three of these reminder messages were created by the study team in order to test the hypotheses set out below; one was the existing reminder message used by the Trust. We hypothesized that these new reminder messages would lead to DNA rates that were significantly lower than those produced by the existing message. To test this hypothesis, records of message assignment were compared against records of whether a DNA subsequently occurred. The Trust recorded a DNA as occurring if a patient did not present at an appointment and did not cancel the appointment in advance. It is important to note that none of the new reminder messages exceeded the 160 character SMS limit, and therefore costs were kept constant.

### Interventions

Table 1 sets out the text of the messages. The then current reminder message was retained as the control intervention. The study team created three new reminder messages: “Easy Call”, “Social Norms”, and “Specific Costs”. The main purpose of the “Easy Call” message was to include the telephone number for cancelling appointments in the body of the SMS reminder, as opposed to referring the patient to their appointment letter. This change was based on the well-established findings that even small reductions in the effort required to perform an action can have a significant impact on behaviour [17].

**Table 1:** Messages for Trial One.

Message	Wording
**Control**	Appt at [clinic] on [date] at [time]. To cancel or rearrange call the number on your appointment letter.
**Easy Call**	Appt at [clinic] on [date] at [time]. To cancel or rearrange call 02077673200.
**Social Norms**	We are expecting you at [clinic] on [date] at [time]. 9 out of 10 people attend. Call 02077673200 if you need to cancel or rearrange.
**Specific Costs**	We are expecting you at [clinic] on [date] at [time]. Not attending costs NHS £160 approx. Call 02077673200 if you need to cancel or rearrange.

The “Social Norms” message communicated the fact that the dominant social norm was to attend an appointment. There is evidence from other fields that people overestimate the extent to which others perform acts that are non-optimal (or which cause harm to others), and that correcting these perceptions can change behaviour [18] [19]. Further, the specific tactic of referring to high attendance rates has been shown to reduce DNAs in a primary care setting (although using different media) [20]. Note that we retain the telephone number in this and subsequent variations.

The “Specific Costs” message noted the cost of a missed appointment. The patient may not be aware that missing an appointment incurs a cost. Even if they are, the cost may not be salient, since it is likely to be seen as just an “opportunity cost”–i.e. the loss of an opportunity to do something more productive [21]. There is emerging evidence that making such costs salient may influence behaviour. For example, giving clinicians feedback on the cost of discretionary diagnostic tests influences subsequent demand for tests [22]. Therefore, there are good reasons to examine the effect of presenting the potential cost of a missed appointment. To identify the cost figure, we obtained the 2012–13 NHS tariff for initial and follow-up appointments in all five specialities. Across all categories, the mean cost was £161. To reflect the fact that this was a simple average, it was appropriate to represent the figure as an approximation.

### Power calculations

Our decision to create four treatment groups was informed by a power analysis. On the basis of past appointment data, we calculated that the five specialities dealt with approximately 17,000 appointments per month. We also observed that SMS messages were being issued for approximately 20% of appointments in the specialities, meaning that around 3,400 appointments would fall into the sample each month. On the basis of previous message-based studies, we aimed to detect a 1.5 percentage point change in the DNA rate (representing 0.16 standard deviations) with 80% power. These calculations indicated that three months would be required to collect the 10,000 cases needed to obtain this power.

### Randomisation

The randomisation was conducted as follows. The SMS provider iPlato applied a Mersenne Twister computer-generated randomisation syntax to automatically allocate appointments to reminder messages [23]. This was a simple randomisation procedure with no blocking. None of the researchers or hospital staff took part in the randomisation or were able to access allocation details until the data were provided at the end of the trial. Randomisation was applied to appointments, rather than to participants, which means that participants could receive the same or different reminder(s) over the course of the study.

### Analysis

To analyse the main trial outcomes, we applied a binary logistic regression model using STATA version 13. In line with our hypothesis, the model took the message groups as treatment variables, with the existing message used by the Trust as the constant or reference group. The presence or absence of a DNA, as recorded by the Trust, was the binary outcome variable. We added in age and gender to the model, and interacted these factors with the different message treatments. We also conducted subgroup analysis by speciality, although the study was not powered for this analysis. We conducted secondary analyses in order to assess the impact of the messages on levels of appointment attendance and patient cancellation, although we did not have directional hypotheses for these outcomes. These secondary analyses consisted of two further binary logistic regressions, one with appointment attendance as the binary outcome variable, and one with patient cancellation as the binary outcome variable. Finally, we conducted a chi squared omnibus test on our three non-control messages, in order to determine if their combined effect was statistically significant.

## Trial One Results

**Fig 1:**
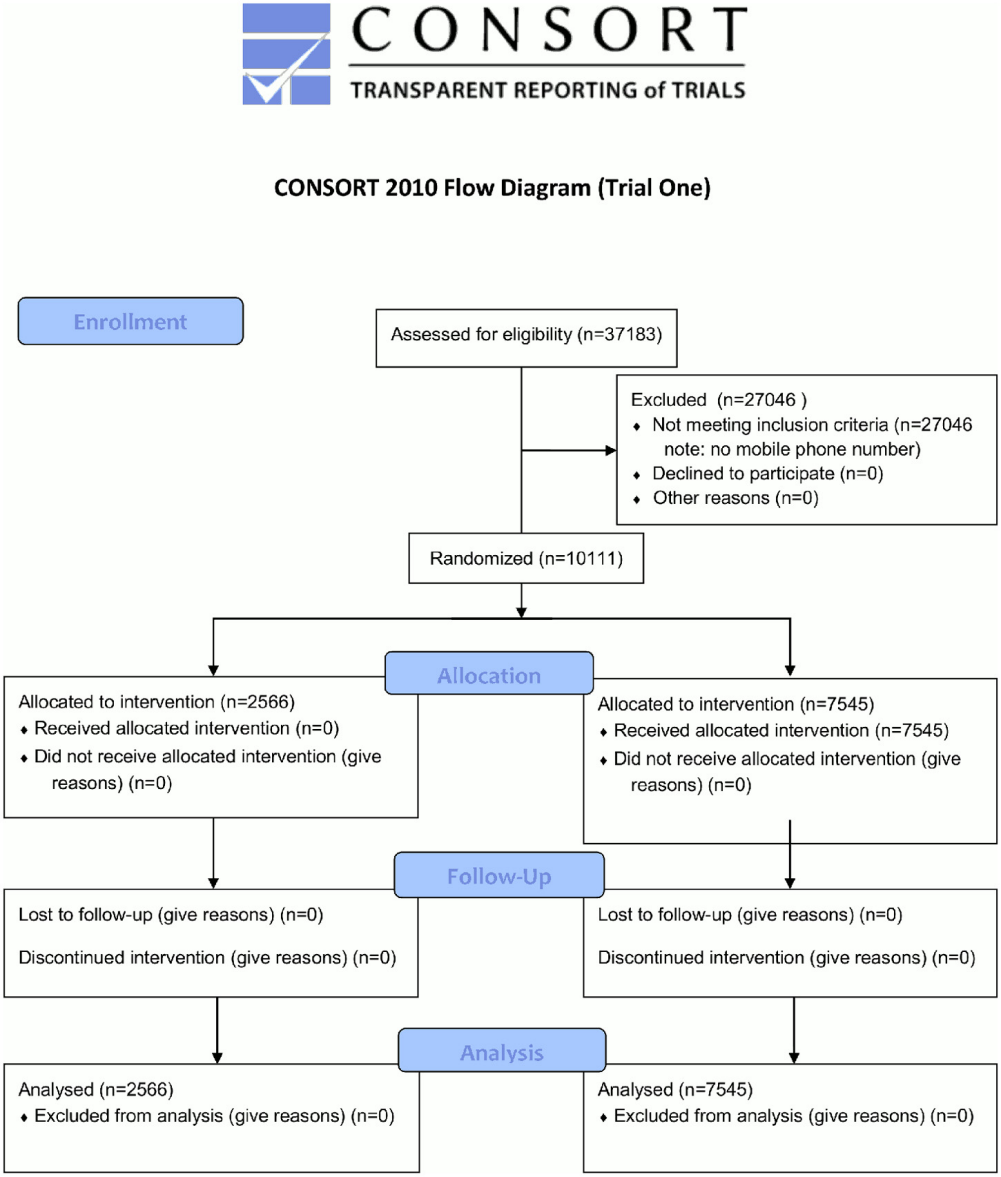
CONSORT flow diagram for Trial One.

**Fig 2:**
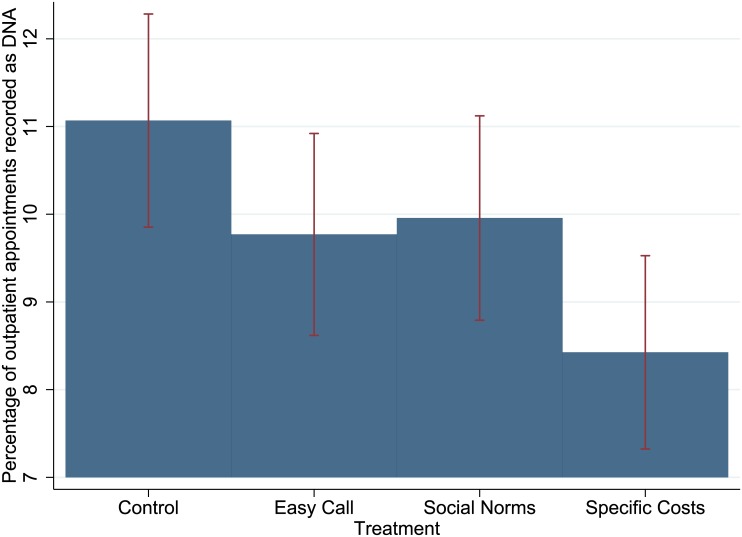
Main results for Trial One. The Specific Costs message produced a DNA rate of 8.4%, compared to a rate of 11.1% for the Control message (OR 0.74, 95% CI 0.61–0.89, P<0.01). None of the other messages had a significant effect on this outcome measure.

Of the 37,183 appointments occurring in the specialities during Trial One, 10,111 had mobile phone records that permitted an SMS reminder to be sent (Fig 1). Table 2 gives the results for the main trial outcomes in raw percentages, alongside key descriptive statistics. Table 3 gives the regression outputs for the primary and secondary analyses. The Specific Costs message produced a DNA rate of 8.4% (206/2445), compared to a rate of 11.1% (284/2566) for the Control message (OR 0.74, 95% CI 0.61–0.89, P<0.01). No other messages had a significant effect on DNA rates. Fig 2 shows these results as a graph. Interestingly, the Social Norms message led to more appointment cancellations (Control 8.8%, 225/2566, Social Norms 10.5%, 268/2541, OR 1.23, 95% CI 1.02–1.48, P = 0.03).

**Table 2:** Trial One main results (raw data).

	Main results			Descriptive statistics			
	DNA	Attend	Cancel	N	Male	Female	Age (Mean)
**Existing message (control)**	11.1%	77.6%	8.8%	2566	39.7%	60.3%	48.7
**Easy Call**	9.8%	77.7%	9.7%	2559	40.1%	59.9%	49.2
**Social Norms**	10.0%	76.7%	10.1%	2541	39.2%	60.8%	48.9
**Specific Costs**	8.4%	78.9%	9.6%	2445	40.7%	59.3%	48.7

Percentage figures do not sum to 100 because it was also possible for the hospital to cancel an appointment. This outcome has been omitted because it is not relevant to the current study.

**Table 3:** Trial One main results (multivariate logistic regression).

		Regression I: DNA	Regression II: Attendance	Regression III: Cancellation
**Intervention effects (Reference: Control message)**	**Easy Call**	0.87	1.01	1.12
		[0.73, 1.04]	[0.88, 1.15]	[0.92, 1.35]
	**Social Norms**	0.89	0.95	1.23*
		[0.74, 1.06]	[0.84, 1.09]	[1.02, 1.48]
	**Specific Costs**	0.74**	1.09	1.10
		[0.61, 0.89]	[0.95, 1.24]	[0.91, 1.33]
	***N***	10111	10111	10111
**Intervention effects with covariates (Reference: Control message)**	**Easy Call**	0.87	1.01	1.12
		[0.73, 1.05]	[0.88, 1.15]	[0.93, 1.35]
	**Social Norms**	0.89	0.95	1.23*
		[0.74, 1.07]	[0.84, 1.09]	[1.02, 1.48]
	**Specific Costs**	0.74**	1.08	1.10
		[0.61, 0.89]	[0.95, 1.24]	[0.91, 1.34]
	**Male**	0.93	1.15**	0.84*
		[0.82, 1.07]	[1.04, 1.26]	[0.736,0.968]
	**Age**	0.99***	1.01***	0.997
		[0.99, 0.10]	[1.00, 1.01]	[0.993,1.001]
	***N***	10111	10111	10111

* *p* < 0.05;

** *p* < 0.01;

*** *p* < 0.001.

Main figures are odds ratios, with 95% confidence intervals in parentheses.

Table 3 shows that the coefficients remain stable after controlling for age and gender, although it is worth noting that these covariates have separate effects on the outcome variables: men are more likely to attend and less likely to cancel; older patients are less likely to DNA and more likely to attend. There are no significant interactions between message treatments and age or gender, and we have not presented these results for reasons of space. Table 4 shows that the results for the DNA outcome are sustained across the five different specialities. Furthermore, including specialities as covariates in the regression does not affect the main results. Finally, the chi squared omnibus test on our three non-control messages produced a statistically significant result at the 0.05 level (p = 0.02).

**Table 4:** Trial One DNA results by speciality.

Intervention effects (Reference: Control message)	Cardiology	Gastroenterology	Neurology	Ophthalmology	Rheumatology
**Easy Call**	0.64	0.82	0.51	1.01	0.96
	[0.38, 1.09]	[0.56, 1.20]	[0.17, 1.50]	[0.65, 1.57]	[0.73, 1.26]
**Social Norms**	0.82	0.76	0.65	0.88	1.01
	[0.50, 1.35]	[0.52, 1.13]	[0.25, 1.65]	[0.56, 1.40]	[0.77, 1.32]
**Specific Costs**	0.56*	0.85	0.16*	0.96	0.73*
	[0.32, 0.99]	[0.58, 1.24]	[0.03, 0.71]	[0.60, 1.52]	[0.54, 0.98]
***N***	1404	2197	331	1446	4534

* *p* < 0.05.

Main figures are odds ratios, with 95% confidence intervals in parentheses.

## Trial Two Methods

Following the results from Trial One, we amended our original trial protocol to allow us to conduct a second trial, which built on the results described above. This trial took place in March, April and May 2014. All other aspects of the trial design and methods are identical to Trial One. We carried out the same analytic procedures as in Trial One, with the exception that the Specific Costs message (i.e. the best-performing message from Trial One) was used as the control intervention.

Table 5 gives the four messages included in Trial Two. As noted, this trial retained the Specific Costs message, while introducing three new messages: “General Costs”, “Empathy” and “Recording”. The “General Costs” message was constructed to present the concept of costs in more general terms. Creating a more general message is attractive for practical reasons, since it requires less effort to adopt at scale (specific costs do not have to be calculated). However, studies have shown that tailored messages are more likely to attract attention and trigger conscious thought [24] [25]. We therefore hypothesized that the General Costs message would be less effective at preventing DNAs than the Specific Costs message.

**Table 5:** Trial Two messages.

Message	Wording
**Specific Costs**	We are expecting you at [clinic] on [date] at [time]. Not attending costs NHS £160 approx. Call 02077673200 if you need to cancel or rearrange.
**General Costs**	We are expecting you at [clinic] on [date] at [time]. Not attending wastes NHS money. Call 02077673200 if you need to cancel or rearrange.
**Empathy**	We are expecting you at [clinic] on [date] at [time]. Please be fair to others waiting and call 02077673200 if you need to cancel or rearrange.
**Recording**	We are expecting you at [clinic] on [date] at [time]. Please attend or call 02077673200 to cancel/rearrange, or we will record as a missed appt.

The “Empathy” message reminded the recipient of the situation of others. There is much evidence that situationally-induced empathy (asking someone to “put yourself in others’ shoes”) leads to an increase in pro-social behaviour [26] [27]. In this case, missing an appointment often means unnecessary delay to the treatment of others, even if some form of overbooking is taking place. The final “Recording” message simply pointed out that the hospital records missed appointments. Evidence from other fields shows that increasing “visibility” of an individual’s actions through creating data records (and notifying them of that fact) increases pro-social behaviour [28] [29]. We did not have a directional hypothesis for the effect of the Empathy and Recording messages compared to the Specific Costs message.

## Trial Two Results

**Fig 3:**
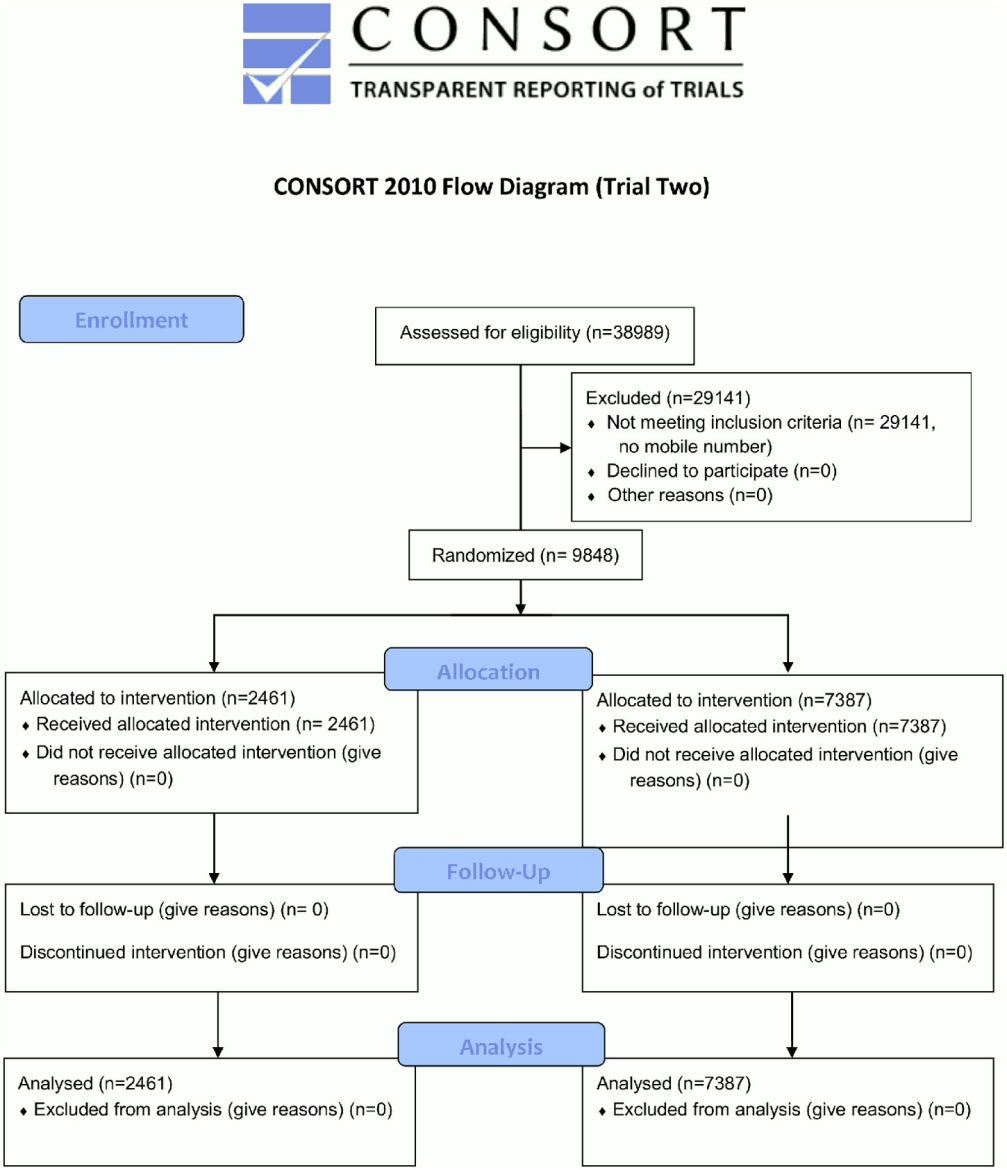
CONSORT flow diagram for Trial Two.

Of the 38,989 appointments occurring in the specialities during Trial Two, 9,848 had mobile phone records that permitted an SMS reminder to be sent (Fig 3). Table 6 gives the results for the main trial outcomes in raw percentages alongside key descriptive statistics, while Table 7 gives the regression outputs. Trial Two replicates the Specific Costs result from Trial One, in that it produces a DNA rate (8.2%, 203/2461) that is similar in absolute terms to the result from the previous trial, while also being significantly lower than those resulting from the two of the three other messages tested.

**Table 6:** Trial Two main results and descriptive statistics (raw data).

Intervention	Main results			Descriptive statistics			
	DNA	Attend	Cancel	N	Male	Female	Age (Mean)
**Specific Costs (control)**	8.2%	79.0%	9.5%	2461	42.5%	57.5%	48.3
**General Costs**	9.9%	78.5%	9.2%	2553	39.3%	60.7%	48.8
**Empathy**	10.7%	76.2%	10.8%	2439	41.1%	58.9%	49.1
**Recording**	9.6%	77.1%	10.2%	2395	41.6%	58.4%	48.7

Percentage figures do not sum to 100 because it was also possible for the hospital to cancel an appointment. This outcome has been omitted because it is not relevant to the current study.

**Table 7:** Trial Two main results and covariates (multivariate logistic regression).

		Regression I: DNA	Regression II: Attendance	Regression III: Cancellation
**Intervention effects (Reference: Specific Costs message)**	**General Costs**	1.22*	0.97	0.96
		[1.00, 1.48]	[0.85, 1.11]	[0.79,1.16]
	**Empathy**	1.33**	0.85*	1.14
		[1.10, 1.62]	[0.75, 0.98]	[0.95, 1.37]
	**Recording**	1.18	0.89	1.069
		[0.97, 1.44]	[0.78, 1.02]	[0.89, 1.29]
	***N***	9848	9848	9848
**Intervention effects with covariates (Reference: Specific Costs message)**	**General Costs**	1.23*	0.96	0.96
		[1.01, 1.49]	[0.84, 1.10]	[0.79, 1.16]
	**Empathy**	1.35**	0.85*	1.14
		[1.11, 1.63]	[0.74, 0.97]	[0.95, 1.38]
	**Recording**	1.19	0.89	1.07
		[0.97, 1.45]	[0.78, 1.02]	[0.89, 1.29]
	**Male**	1.16*	0.97	0.90
		[1.01, 1.33]	[0.88, 1.07]	[0.79, 1.03]
	**Age**	0.99***	1.01***	0.99**
		[0.99, 0.99]	[1.01, 1.01]	[0.99, 1.0]
	***N***	9848	9848	9848

* *p* < 0.05;

** *p* < 0.01;

*** *p* < 0.001.

Main figures are odds ratios, with 95% confidence intervals in parentheses. Exponentiated coefficients.

**Fig 4:**
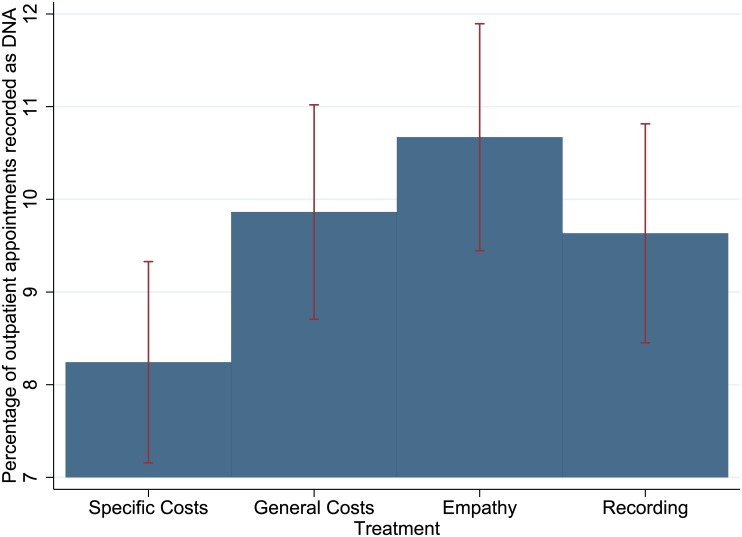
Main results for Trial Two. The General Costs message was less effective at reducing DNAs than the Specific Costs message: it produced a DNA rate of 9.9%, compared to 8.2% for the Specific Costs message (OR 1.22, 95% CI 1.00 to 1.48, P = 0.046).

As hypothesized, the General Costs message was less effective at preventing DNAs than the Specific Costs message: it produced a DNA rate of 9.9% (252/2553), compared to 8.2% for the Specific Costs message (OR 1.22, 95% CI 1.00–1.48, P = 0.046). The Empathy message was also significantly less effective than the Specific Costs message (10.7%, 261/2439, OR 1.33, 95% CI 1.10–1.62, P<0.01). The Recording message produced a DNA rate of 9.6% (230/2395), which is not significantly higher than the Specific Costs message, although it was close to significance at the 0.05 level (OR 1.18, 95% CI 0.97–1.44, P = 0.07). Fig 4 shows the main treatment effects in a graph. Table 7 shows that there are negligible changes to the results after controlling for age and gender, although it is worth noting that these covariates have separate effects on the DNA variable: men and younger patients are significantly more likely to DNA. There are no significant interactions between message treatments and age or gender, and therefore we have not presented these results. As Table 8 shows, the overall pattern of results is sustained when the data are analysed by speciality, with the exception of Neurology (the smallest group). Furthermore, including specialities as covariates in the regression does not affect the main results. Finally, a chi squared omnibus test on our three non-control messages produced a statistically significant result at the 0.05 level (p = 0.032).

**Table 8:** Trial Two, DNA results by speciality.

Intervention effects (Reference: Specific Costs message)	Cardiology	Gastroenterology	Neurology	Ophthalmology	Rheumatology
**General Costs**	1.55	1.45	0.69	0.90	1.23
	[0.83, 2.89]	[0.99, 2.13]	[0.24, 2.0]	[0.56, 1.44]	[0.92, 1.650]
**Empathy**	2.12*	1.30	1.21	1.16	1.29
	[1.16, 3.85]	[0.88, 1.94]	[0.45,3.26]	[0.73, 1.84]	[0.97, 1.73]
**Recording**	1.15	1.03	0.36	1.62*	1.18
	[0.59, 2.25]	[0.68, 1.56]	[0.09, 1.43]	[1.05, 2.50]	[0.88, 1.59]
***N***	1335	2333	219	1550	4411

* *p* < 0.05.

Main figures are odds ratios, with 95% confidence intervals in parentheses.

## Discussion

These results show that the wording of SMS reminders significantly affects the extent to which patients miss, attend or cancel outpatient appointments. Moreover, the results show that presenting the specific tariff cost of the appointment produces a DNA rate that is approximately three percentage points lower than for other messages, a result that we replicate. The fact that there were no significant differences between the Control and Social Norms messages means we can be confident that the effect of the Specific Costs is not being driven by the “We are expecting you” message framing, or the presence of the telephone number. The changes were not anticipated to produce adverse effects for patients, not least because they retained the freedom to attend, cancel or not attend their appointment. Based on our analysis of the available outcome data, there is no reason to believe that harms had occurred. It is worth noting that the Social Norms message significantly increased the level of patient cancellations; however, we cannot interpret this as a poor outcome that increases harm, since a patient cancellation is one route to avoiding a missed appointment and attending at a future date.

The results indicate that the wording of reminders can reduce DNA rates by around a quarter in relative terms. These treatment effects should be placed in context. As already noted, in Trial Two the odds ratio between the Specific Costs and Empathy messages was 1.33 (95% CI 1.10–1.61). This compares favourably with the summary effect of 1.48 for SMS reminders *per se* reported by a recent meta-analysis [30] [11]. In crude terms, this comparison suggests that the content of SMS reminders may constitute a large part of their overall effect. The more general implication is that health system performance improvements do not depend on technical solutions alone.

Newly-worded messages constituted the sole element of this intervention; no other aspects of patient care were altered. It should be noted that the reminders were sent a relatively long time before the appointment (five days) compared to those in previous studies, which were generally sent between one and three days in advance [10]. Having a relatively long period between the message and appointment is likely to increase the possibility that the reminder effect decays in that period. Ideally, we would have issued the messages one to three days in advance, but we were not able to alter the existing schedule.

These results have clear policy implications. We calculate that the lower DNA rate produced by the Specific Costs message (compared to the Control message) in Trial One would result in 5,800 fewer missed appointments for Barts Health across all outpatient specialities, if sustained over the course of a year. This figure was obtained by extrapolating the total number of appointments from the six months of available data to estimate the total number of appointments for a year. The 2.6% marginal effect was then applied to this estimated total. The estimated number of appointments is based on a mobile phone coverage rate of 20%, and thus it may be conservative figure. On the same basis, full phone record coverage could result in 28,900 fewer missed appointments for the Trust annually. It also worth noting that a 2.6 percentage point change in the DNA rate across all outpatient appointments in England (assuming 20% mobile phone coverage) equates to 400,000 fewer DNAs annually. However, we wish to stress that this estimate is dependent on several factors, particularly on the extent to which the result generalizes to other specialities, and the volume of appointments in each speciality. Since these figures are extrapolations they should be treated with caution.

The wider implications of this trial are that healthcare providers with SMS technology looking to reduce DNAs should consider adopting the messages above, or testing their own wordings. More generally, there is evidence that they should ensure messages contain specific, salient information in order to maximise their effectiveness. If the 160 character limit is not exceeded, then there is no additional cost to such a change. In addition, improving mobile phone records (to extend the use of SMS messages) may be an inexpensive way of reducing DNAs.

These trials have several limitations. Scaling up the use of these messages is dependent on Trusts possessing accurate records of mobile telephone numbers. As noted above, SMS reminders were only issued to 20% of individuals with appointments in the study period. We cannot observe whether the populations with or without accurate mobile number telephone records differ systematically from each other. We cannot rule out the possibility that the population who received the SMS reminders may have been more sensitive to the intervention than those who did not (which is another reason to be cautious of the extrapolations above). The study team were also unable to access records about the proportion of messages that were actually read or received, although the randomised nature of the study means we can retain confidence in our overall conclusions.

In addition, it is uncertain how far the effect of these messages can be replicated in different regions and different health systems. The trial took place in a London Trust. London sees higher DNA rates than other parts of the UK, which means that the effect of these interventions may be smaller in those areas [2]. However, it should be noted that the best-performing message produced a DNA rate of 8.2%, which is below the 9.3% average rate for England. More broadly, it is unclear whether mentioning costs to the health system is an effective strategy in health systems with different funding structures from that of the NHS. The generalizability of such messages is a pressing area for future research.

## Supporting Information

S1 CONSORT Checklist (DOC)

S1 Data Expanded Dataset for Trial One. (XLS)

S2 Data Expanded Dataset for Trial Two. (XLS)

S1 Protocol Trial protocol. (DOCX)
