# Correction: Stating Appointment Costs in SMS Reminders Reduces Missed Hospital Appointments: Findings from Two Randomised Controlled Trials

**DOI:** 10.1371/journal.pone.0141461

**Published:** 2015-10-20

**Authors:** Michael Hallsworth, Dan Berry, Michael Sanders, Anna Sallis, Dominic King, Ivo Vlaev, Ara Darzi

The affiliation for the sixth author is incorrect. Ivo Vlaev is not affiliated with #1, but with Warwick Business School, University of Warwick, Coventry, United Kingdom.
