# Supplementary material for: Stating Appointment Costs in SMS Reminders Reduces Missed Hospital Appointments: Findings from Two Randomised Controlled Trials
Source: PLoS One. 2015 Sep 14;10(9):e0137306. doi: 10.1371/journal.pone.0137306 (PMC4569397; doi:10.1371/journal.pone.0137306)
Supplement: S1 Protocol — (DOCX) [file pone.0137306.s004.docx]

**SMS reminder message to reduce missed appointments**

**Research proposal summary: June 2013**

**REC Reference: 13 / NW / 0508**

**Summary**

This study is to test the content of the most effective SMS reminder message to reduce missed appointments in outpatient clinics.

Over 6 million out-patient appointments are missed each year in the NHS (about 8.5% of the total). Non-attendance results in economic loss, administrative problems and poor patient care. In 2008 it was estimated that missed outpatient appointments cost the NHS around £600 million a year.

A systematic review of telephone and SMS reminders found that they significantly improved attendance. A Cochrane Review on the topic found relatively little robust evidence so far, but that which does exist indicates that SMS reminders offer a flexible, effective, cost-effective means of increasing appointment attendance.

While many NHS organisations already use SMS reminders, we believe they can be made more effective by improving the messages they contain. However, there is currently no evidence for which text messages are most effective

We propose to collaborate with Barts Hospital to test different text message invites and reminders to reduce missed appointments.

These messages will draw on the latest evidence of how message content can influence behaviour. We believe this is an opportunity for Barts Hospital – and other hospitals – to improve outcomes at little or no additional cost.

**Working with Barts Health NHS Trust**

We have developed this proposal in conjunction with Greg Bird, outpatients’ manager at Barts Hospital.

No research activity will take place on Barts premises *per se*. iPlato (Barts Health’s outsourced SMS IT provider) will integrate a computer-based randomisation syntax into the system that issues SMS reminder to patients attending an outpatient appointment. There is no change to patients’ standard clinical care

The nature of the intervention we propose to test means this is low-cost research. This is because it uses the text message system already in place at Barts.

The research is led by the behavioural insights teams at the Department of Health and Cabinet Office. They will support the research. Limited time and zero costs are required from Barts.

The only additional cost of the research is a randomisation syntax implemented by iPlato, at no cost to Barts.

**Ethical considerations**

We are concurrently seeking REC approval for this study. This is accepted for a proportionate review. Indeed, we are advised that this is on the cusp of ‘service evaluation’ rather than ‘research’, according to the NRES definition.

We can confirm that the researchers will not have access to any patient identifiable information.

We will receive anonymous and aggregated data detailing the missed appointment rates in each clinic and linked to each of the different SMS messages. We will receive basic demographic data, such as gender and age, but at an aggregated level.

This data will be collected by Barts hospital and their IT supplier, iPlato, in line with their established information governance agreement. This agreement complies with guidelines from the Trust’s Caldicott Guardians as well as legal requirements.

We have considered the impact of patients receiving text messages that, we hypothesise, will be more effective than the current message used by Barts (based upon theoretical and empirical evidence in other areas of the behaviour change literature). We do not believe that the content of the messages will have a negative impact on patients’ health or well-being. There is currently no evidence for which text messages are most effective and, in the absence of this information, hospitals continue to use a wide variety of untested approaches. This research will help create an evidence-based approach.

**Interventions to test and their justification**

Control: “Appt at <clinic> on <date> at <time>. To cancel or rearrange call the number on your appointment letter.”

We propose to retain this current SMS message as a control while introducing the following variants

Trial arm A “Appt at <clinic> on <date> at <time>. To cancel or rearrange call 0207389471.”

Reducing even apparently minor barriers to carrying out a behaviour can significantly increase the incidence of that behaviour. For example, a seminal study from the field of social psychology found that vaccination uptake was low even after education had been provided and broad intentions formed. However, uptake increased from (3% to 28%) by simply providing a map and asking participants to think about an appointment time: these small “channel factors” made a large difference (Leventhal, Singer, and Jones, 1965).

Similarly, it is likely that the process of having to locate the appointment letter reduces the effectiveness of the control message. We have been advised that it is possible to include the specific telephone number, and we would like to test the impact of doing so:

Trial arm B “We are expecting you at <clinic> on <date> at <time>. Nine out of ten people attend. Please call 0207389471 if you need to cancel or rearrange.”

There is much evidence that even short messages based on social norms – saying what other people in the same situation - can have a significant effect on compliance (e.g. Hallsworth et al., 2013). Given that only around 10% of patients do not attend, there is a clear opportunity to leverage social norms:

Trial arm C “We are expecting you at <clinic> on <date> at <time>. Not attending costs NHS £160 on average, so call 0207389471 if you need to cancel or rearrange.”

Given that people are exposed to a great amount of information every day, a message must be framed in a salient way to have the best chance of being effective (Dolan et al., 2010). The costs of missing an appointment are likely to be unknown to the patient. Alternatively, he/she may have some awareness of the “opportunity cost” incurred by a missed appointment, but this is likely to be of very low salience (Frederick et al. 2009). Therefore, stating these costs in a salient way is likely to improve the effectiveness of the message. Costs vary, but we have established £160 as the average cost for the clinics we propose to work with at Barts.

**Research design and analysis**

This is a between groups randomised controlled trial with (ideally) four trial arms.

The trial is planned to run at Barts Hospital, part of Barts Health NHS Trust, for around 4 months. This location was selected because Barts have an IT system in place that enables randomisation of messages at minimal additional cost and effort.

Five clinics of different specialities have been purposively selected on the basis of high DNA rates and high patient numbers to optimise study sample size. Patients at each clinic are sent appointment letters prior to their outpatient appointment. Patients are then sent a reminder text message five days prior to their appointment.

Patients’ will be randomised (within each clinic) to receive one of the SMS reminder, messages including a message acting as the control which reflects usual practice.

Previous research indicates that this trial needs to be powered to detect effects of 1.5 percentage points with 80% power.

The trial is scheduled to run for four months and we propose 4 arms. We calculate that this would result in approximately 8,800 cases in total. Power calculations suggest that this will allow us to detect a 1.4 percentage points difference in missed appointment rates with 80% power.

We recognise that practical delays or seasonal variations may reduce the number of cases entering the experiment. If such delays occur, we will reduce the number of arms to three in order to ensure we have at least 8,800 cases in total.

We have arranged for the SMS IT provider, iPlato, to integrate computer-based randomisation syntax into the system that issues SMS reminders.

Analysts from the project team will undertake a logistic regression analysis that estimates the effects of each message variant on appointment attendance This analysis will include the various clinics as fixed effects in the regression model, as well as demographic covariates (where available).

**Research Team**

The Department of Health set up a Behaviour Change Team in December 2012. Together with clinical academics at Imperial College London and the Behavioural Insights Team at Cabinet Office, we have formed a working group to apply behavioural insights to tackle the costly problem of DNAs. The main opportunity we have identified is to vary the message content of SMS reminders.

**Contact details for further information**

*Research team*

- Anna Sallis: [anna.sallis@dh.gsi.gov.uk](mailto:anna.sallis@dh.gsi.gov.uk)
- Dan Berry: [Daniel.berry@dh.gsi.gov.uk](mailto:Daniel.berry@dh.gsi.gov.uk)
- Michael Hallsworth: [m.hallsworth11@imperial.ac.uk](mailto:m.hallsworth11@imperial.ac.uk)

*Barts*

- Greg Bird: [Gregory.Bird@bartshealth.nhs.uk](mailto:Gregory.Bird@bartshealth.nhs.uk)
- Mark Nkrumah, Research Approvals Advisor: [mark.nkrumah@bartshealth.nhs.uk](mailto:mark.nkrumah@bartshealth.nhs.uk)

**Behaviour Change Team**

Department of Health

21 June 2013

**Annex 1: The scale of the problem, and potential solutions**

**The problem**

An outpatient appointment takes place when a GP refers a patient to a hospital for a medical problem. The hospital will send a letter confirming the consultant, the location of the clinic and the time of the appointment.

Over 6 million out-patient appointments are missed each year in the NHS (about 8.5% of the total). Non-attendance at outpatient clinics results in economic loss, administrative problems and poor patient care. In 2008 it was estimated that missed hospital outpatient appointments cost NHS hospitals around of £600 million a year.

**Reasons for non-attendance**

The reasons for missing appointments given by patients include (Neal 2005):

- Forgetting the appointment;
- Fear and anxiety;
- Difficult of changing or cancelling the appointment.

Socio-economic and health factors are also associated with missing an appointment. Men in their early 20s most commonly miss their appointments, while the likelihood of missing an appointment increases with levels of deprivation (Dr Foster 2010).

**Strategies to increase attendance**

Many of the strategies for increasing attendance incur significant implementation costs. In contrast, one promising yet relatively simple approach is to remind patients about their hospital appointment through SMS messages. A systematic review of telephone and SMS reminders found that they significantly improved attendance (Hasvold 2011). A Cochrane Review on the topic found relatively little robust evidence so far, but that which does exist indicates (Car et al. 2012):

- SMS reminders are more effective than no reminders;
- SMS reminders with postal reminders are more effective than postal reminders alone;
- SMS reminders are as effective as phone call reminders.

Another Cochrane Review on SMS messages for behaviour change in general concluded that they have positive short-term behavioural outcomes (Fjeldsoe et al. 2009). In sum, SMS reminders may offer a flexible, effective, cost-effective means of increasing appointment attendance.

**Applying behavioural insights**

While many hospitals already use SMS reminders, we believe they can be made more effective by improving the messages they contain.

There is much evidence that the applying behavioural insights to the wording of requests can increase their effectiveness, both in laboratory and real-world settings. For example, members of the working group developed the MINDSPACE framework (Dolan et al. 2012), which consists of nine of the most robust factors that influence behaviour and can be used to inform message design. This framework has been successfully applied to various real-world policy problems, including health (Behavioural Insights Team 2011); fraud and error (Behavioural Insights Team 2012); and energy consumption (Behavioural Insights Team 2011).

Three studies in particular show the power and relevance of this approach.

- **SMS wording affects weight loss.** Two members of the working group recently demonstrated that SMS messages that created commitments (the “C” in the MINDSPACE acronym) were more effective for achieving weight loss than those that simply provided information (Kulendran et al. 2013).
- **Short messages increase tax compliance.** Two members of the working group have run a set of field studies which demonstrate that short messages based on behavioural insights significantly increase real-world tax compliance (Hallsworth et al. 2013).
- **Simple interventions reduce DNAs.** A recent study found that simple interventions based on social norms messages and commitment devices reduced DNAs by up to 31% (Martin et al. 2012).

All these studies show that even short messages based on behavioural insights can produce significant benefits.

**Annex 2: References**

- Behavioural Insights Team (2010) Applying behavioural insight to health. London: The Cabinet Office.
- Behavioural Insights Team (2011) Behaviour change and energy use. London: The Cabinet Office.
- Behavioural Insights Team (2012) Applying behavioural insights to reduce fraud, error and debt. London: The Cabinet Office.
- Car, J., Gurol-Urganci, I., de Jongh, T., Vodopivec-Jamsek, V., Atun, R. (2012) Mobile phone messaging reminders for attendance at healthcare appointments. Cochrane Database Syst Rev., July 11.
- Dolan, P., Hallsworth, M., Halpern, D., King, D. Metcalfe, R. & Vlaev, I. (2012) Influencing behaviour: The MINDSPACE way. Journal of Economic Psychology, 33, 264-277.
- Dr Foster Health. (2010). Out patient no-shows cost hospitals £600m a year. <http://www.drfosterhealth.co.uk/features/outpatient-appointment-no-shows.aspx>
- Fjeldsoe, B., Marshall, A., Miller, Y. (2009) Behavior Change Interventions Delivered by Mobile Telephone Short-Message Service. American Journal of Preventive Medicine, 36 (2), 165-173.
- Frederick, S., Novemsky, N., Wang, J., Dhar, R., & Nowlis, S. (2009) Opportunity cost neglect. Journal of Consumer Research, 36 (4), 553-561.
- Hallsworth, M., List, J., Metcalfe, R., Vlaev, I. (2013) Using norms and fairness concerns increases tax payments. Unpublished working paper, Imperial College London.
- Hasvold, P.E. & Wootton, R. (2011) Use of telephone and SMS reminders to improve attendance at hospital appointments: a systematic review. Journal of Telemedicine & Telecare, 17, 358 - 364.
- Kulendran, M., Vlaev, I., King, D., Gately, P., Darzi, A. (2013) The use of commitment text messages to maintain weight loss in obese adolescents and the moderating role of impulsivity. Unpublished working paper, Imperial College London.
- Leventhal, H., Singer, R., & Jones, S. (1965) Effects of fear and specificity of recommendation upon attitudes and behavior. Journal of Personality and Social Psychology, 2 (1), 20-29.
- Martin, S., Bassi, S., Dunbar-Rees, R. (2012) Commitments, norms and custard creams – a social influence approach to did not attends (DNAs). Journal of the Royal Society of Medicine, 105 (3), 101-104.
- Neal, H et al. (2005) Reasons for and consequences of missed appointments in general practice in the UK: questionnaire survey and prospective review of medical records, BMC Family Practice, 6.
